# Pulmonary Parenchymal ^18^F-FDG Uptake on PET/MRI in Pulmonary Arterial Hypertension: A Comparative Study with Healthy Controls and Prognostic Implications

**DOI:** 10.3390/jcm15176672

**Published:** 2026-08-28

**Authors:** Remigiusz Kazimierczyk, Piotr M. Szumowski, Stephan G. Nekolla, Łukasz Małek, Piotr Błaszczak, Marta Kosciuk, Janusz Mysliwiec, Karol A. Kaminski

**Affiliations:** 1 Department of Cardiology, Medical University of Bialystok, Curie-Sklodowskiej 24a, 15-276 Bialystok, Poland; martkos@interia.pl (M.K.); fizklin@wp.pl (K.A.K.); 2 Department of Nuclear Medicine, Medical University of Bialystok, Curie-Sklodowskiej 24a, 15-276 Bialystok, Poland; piotrmjs@wp.pl (P.M.S.); janusz.mysliwiec69@gmail.com (J.M.); 3 Department of Nuclear Medicine, Technical University Munich, Ismaninger Str., 81675 Munich, Germany; stephan.nekolla@tum.de; 4 Faculty of Rehabilitation, University of Physical Education, Marymoncka 34, 00-968 Warsaw, Poland; lukasz.a.malek@gmail.com; 5 Department of Cardiology, Cardinal Wyszynski Hospital, Krasnicka Ave 100, 20-718 Lublin, Poland; blaszcz12345@interia.pl; 6 Department of Population Medicine and Lifestyle Diseases Prevention, Medical University of Bialystok, Curie-Sklodowskiej 24a, 15-276 Bialystok, Poland

**Keywords:** pulmonary arterial hypertension, PET/MRI, standardized uptake value, right ventricular-pulmonary arterial coupling, hemodynamics, prognosis

## Abstract

**Background:** Pulmonary arterial hypertension (PAH) is characterized by progressive vascular remodeling and right ventricular (RV) dysfunction. ^18^F-FDG PET/MRI may reveal metabolic alterations in the pulmonary parenchyma and vasculature. We investigated pulmonary FDG uptake in PAH versus healthy controls and its associations with hemodynamics, RV function, and clinical outcomes. **Methods:** Twenty-eight stable PAH patients and 12 age-matched healthy controls underwent ^18^F-FDG PET/MRI. Standardized uptake values (SUVs) were measured in lung parenchyma and proximal pulmonary arteries. Hemodynamic parameters were obtained via right heart catheterization; RV–PA coupling was assessed as stroke volume/end-systolic volume (SV/ESV). Twenty PAH patients underwent follow-up imaging after targeted therapy. Clinical endpoints (CEPs: death, hospitalization, disease progression) were analyzed by Kaplan–Meier and Cox regression. **Results:** PAH patients showed markedly elevated lung parenchymal SUV (0.405 [0.338–0.533] vs. 0.225 [0.207–0.273], *p* < 0.001) and proximal PA SUV (3.46 [1.95–6.89] vs. 1.48 [1.15–1.65], *p* < 0.001). The two metrics were uncorrelated (r = +0.105, *p* = 0.595). SUV PA Proximal correlated with mPAP (r = +0.551) and PVR (r = +0.517), while SUV Lung showed no hemodynamic correlations. After 24 months of therapy, RV–PA coupling improved significantly (*p* = 0.037); lung SUV showed a non-significant trend toward reduction (Δ = −0.10, *p* = 0.128). Sixteen CEPs occurred; impaired RV–PA coupling (HR = 0.04, *p* = 0.002), elevated mPAP (HR = 1.08, *p* < 0.001), and reduced RVEF (HR = 0.91, *p* < 0.001) were strong univariable predictors. Neither SUV metric retained independent prognostic value in multivariable analysis. **Conclusions:** Pulmonary parenchymal and proximal PA FDG uptake are markedly elevated in PAH. However, neither metric independently predicts hemodynamic severity or clinical outcomes, limiting their current role as reliable prognostic surrogates.

## 1. Introduction

Pulmonary arterial hypertension (PAH) is a progressive disorder characterized by pulmonary vascular remodeling, increased pulmonary vascular resistance (PVR), and right ventricular (RV) failure. Despite advances in PAH-targeted therapies, morbidity and mortality remain substantial, underscoring the need for improved diagnostic and prognostic biomarkers [1,2]. Traditional assessments rely on invasive hemodynamic measurements, echocardiography, and functional capacity testing. However, these modalities provide limited insight into the underlying metabolic and cellular processes driving disease progression.

Hybrid positron emission tomography/magnetic resonance imaging (PET/MRI) offers the advantage of simultaneous metabolic and anatomical assessment with superior soft tissue contrast and reduced radiation exposure compared to PET/computed tomography [3]. ^18^F-fluorodeoxyglucose (FDG) PET has emerged as a tool to assess metabolic activity in cardiovascular diseases. We previously confirmed that in PAH, increased FDG uptake in the RV myocardium has been associated with RV dysfunction and adverse outcomes (presented as SUV RV/LV ratio) [4,5]. However, the metabolic signature of the pulmonary parenchyma and vasculature in PAH remains not fully characterized.

The concept of RV-Pulmonary artery (PA) coupling, quantified by the ratio of stroke volume to end-systolic volume (SV/ESV), has gained recognition as a key determinant of RV adaptation to increased afterload and a strong predictor of outcomes in PAH. Impaired coupling reflects maladaptive RV remodeling and is associated with clinical deterioration [6,7,8]. Whether pulmonary metabolic activity, as measured by lung parenchymal FDG uptake, relates to RV-PA coupling or hemodynamic severity remains unknown.

This study aimed to: (1) compare pulmonary lung parenchymal and PA proximal FDG uptake between PAH patients and age-matched healthy controls; (2) investigate associations between both SUV parameters and hemodynamic parameters, RV function, and RV-PA coupling; (3) assess changes in SUV parameters following PAH-targeted therapy; and (4) evaluate the prognostic value of these parameters for event-free survival.

## 2. Materials and Methods

### 2.1. Study Design and Population

This prospective observational study enrolled 28 patients with PAH and 12 age-matched healthy controls between 1 Jun 2018 and 1 Jun 2020. PAH was diagnosed according to (then current) standard hemodynamic criteria (mean pulmonary artery pressure [mPAP] ≥ 25 mmHg, pulmonary capillary wedge pressure [PCWP] ≤ 15 mmHg, and PVR > 3 Wood units) confirmed by right heart catheterization (RHC) [9]. PAH etiologies included idiopathic PAH (iPAH, *n* = 19), connective tissue disease-associated PAH (CTD-PAH, *n* = 5), congenital heart disease-associated PAH (CHD-PAH, *n* = 3), and heritable PAH (hPAH, *n* = 1). Patients were classified by World Health Organization (WHO) functional class: I (*n* = 4), II (*n* = 12), and III (*n* = 12). Healthy controls had no history of cardiovascular or pulmonary disease and normal baseline echocardiography.

All participants provided written informed consent. The study was approved by the institutional review board at Medical University of Bialystok (R-I-002/140/2017) and conducted in accordance with the Declaration of Helsinki.

### 2.2. PET/MRI Acquisition and Analysis

Imaging was performed on a hybrid 3T PET/MRI scanner (Siemens, Munich, Germany). Participants underwent ^18^F-FDG PET/MRI after a minimum 6 h fast. FDG (3–5 MBq/kg) was administered intravenously, followed by a 60 min uptake period as previously described [4,5]. Cardiac MRI sequences included cine imaging for ventricular volumetry and function.

Mean standardized uptake value (SUV), defined as the average SUV within each ROI, was calculated as the ratio of tissue radioactivity concentration to the injected activity normalized to body weight. Regions of interest (ROIs) were manually drawn on fused PET/MRI images to measure SUV mean in: (1) lung parenchyma (avoiding large vessels and airways), and (2) proximal pulmonary arteries [10].

### 2.3. Hemodynamic Assessment

Right heart catheterization was performed within 48 h of PET/MRI. Hemodynamic parameters included systolic, diastolic, and mean pulmonary artery pressures (PAPs, PAPd, mPAP), PCWP, central venous pressure (CVP), cardiac output (CO), cardiac index (CI), and PVR. CO was measured by thermodilution or the Fick method.

### 2.4. RV-PA Coupling Assessment

RV-PA coupling was quantified using the SV/ESV ratio derived from cardiac MRI. RV end-diastolic volume (EDV), end-systolic volume (ESV), and stroke volume (SV) were measured from short-axis cine stacks using standard post-processing software. RV ejection fraction (EF) was calculated as (SV/EDV) × 100%. Tricuspid annular plane systolic excursion (TAPSE) was measured from four-chamber cine images. RV global longitudinal strain (GLS) was assessed using feature-tracking software.

### 2.5. Clinical Follow-Up and Outcomes

PAH patients were followed prospectively for clinical events. The primary endpoint was combined end-point (CEP), defined as time to first occurrence of: all-cause death, lung transplantation, hospitalization for PAH worsening, or initiation of parenteral prostacyclin therapy. Functional capacity was assessed by 6 min walk test (6MWT) distance, and BNP levels were measured at baseline and follow-up after 24 months since enrollment.

A subset of 20 PAH patients underwent repeat PET/MRI and hemodynamic assessment after initiation or escalation of PAH-targeted therapy.

### 2.6. Statistical Analysis

Continuous variables were assessed for normality using Shapiro–Wilk tests. Normally distributed variables are presented as mean ± standard deviation (SD), and non-normally distributed variables as median [interquartile range, IQR]. Categorical variables are presented as counts and percentages.

Between-group comparisons (PAH vs. controls) were performed using Welch’s t-test for normally distributed variables and Mann–Whitney U test for non-normally distributed variables. Paired changes after treatment were analyzed using paired t-tests or Wilcoxon signed-rank tests. Correlations between lung SUV and hemodynamic/functional parameters within the PAH cohort were assessed using Spearman’s rank correlation. Because multiple correlation pairs were tested simultaneously, all correlation *p*-values were adjusted using the Benjamini-Hochberg false discovery rate (FDR) procedure.

Kaplan–Meier survival curves were constructed for CEP, and log-rank tests were used to compare survival distributions stratified by median SUV Lung and SV/ESV. Univariable Cox proportional hazards regression was performed to assess associations between baseline variables and EFS. Multivariable Cox regression included SUV Lung, SV/ESV, and mPAP. Model performance was evaluated using the concordance index (C-index). Statistical significance was set at *p* < 0.05 (two-tailed). Analyses were performed using STATA13 (StataCorp LLC, College Station, TX, USA).

## 3. Results

### 3.1. Baseline Characteristics

**Figure 1:**
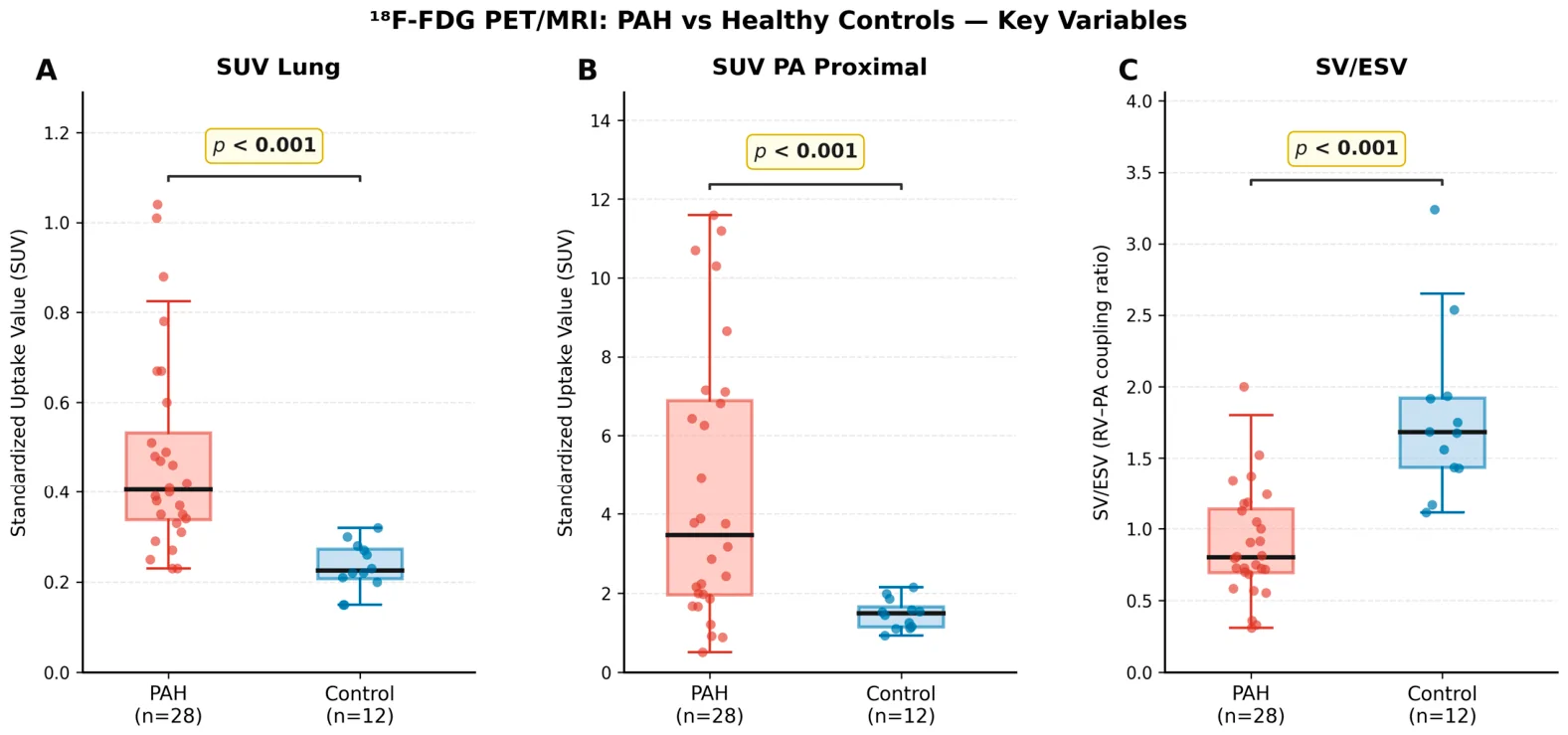
Comparison of key variables between PAH patients and healthy controls. Box plots show distributions of (**A**) SUV Lung, (**B**) SUV PA Proximal, (**C**) SV/ESV (RV-PA coupling). Individual data points are overlaid. ^18^F-FDG; fluorine-18 fluorodeoxyglucose; ESV, end-systolic volume; MRI, magnetic resonance imaging; PA, pulmonary artery; PAH, pulmonary arterial hypertension; PET, positron emission tomography; SUV, standardized uptake value; SV, stroke volume.

The study cohort comprised 28 PAH patients (mean age 51.4 ± 15.9 years) and 12 healthy controls (44.8 ± 13.5 years, *p* = 0.19). Age distributions were well-matched between groups. Among PAH patients, the majority had idiopathic PAH (68%), followed by CTD-PAH (18%), CHD-PAH (11%), and hPAH (4%). Most patients were in WHO functional class II (43%) or III (43%), with 14% in class I. PAH patients demonstrated markedly elevated pulmonary parenchymal FDG uptake compared to healthy controls. Median lung SUV was 0.405 [0.338–0.533] in PAH versus 0.225 [0.207–0.273] in controls (*p* < 0.001). Similarly, proximal pulmonary artery SUV was significantly higher in PAH (3.46 [1.95–6.89] vs. 1.48 [1.15–1.65], *p* < 0.001, r = −0.670). These findings are illustrated in Figure 1.

PAH patients exhibited severe RV dysfunction and remodeling compared to controls (RVEF (44.3 ± 10.1% vs. 63.8 ± 5.9%, *p* < 0.001; RV GLS (−18.0 ± 8.0% vs. −31.5 ± 9.7%, *p* < 0.001). RV-PA coupling, assessed by SV/ESV, was also impaired in PAH (0.80 [0.69–1.14] vs. 1.68 [1.43–1.92], *p* < 0.001). Full baseline characteristics are summarized in Table 1.

### 3.2. Correlations Between SUV and Hemodynamic Parameters

Within the PAH cohort, lung SUV did not correlate significantly with any hemodynamic or functional parameter (Table 2). The strongest trend was observed with pulmonary trunk diameter (Spearman r = −0.35, *p* = 0.064). SUV PA Proximal demonstrated robust correlations with multiple invasive hemodynamic parameters and cardiac biomarkers. Specifically, with mPAP (r = +0.551, *p* = 0.002) and PVR (r = +0.517, *p* = 0.005). Furthermore, tertile analysis revealed a clear gradient of hemodynamic severity across SUV PA Proximal strata: patients in the low tertile had a median mPAP of 34 mmHg and PVR of 4.35 Wood units, whereas those in the high tertile had a median mPAP of 55.5 mmHg and PVR of 11.62 Wood units (Kruskal–Wallis *p* = 0.042).

It is also worth mentioning that there was also no correlation between both SUV parameters and RV-PA coupling (SV/ESV) in PAH patients. When patients were stratified into tertiles of RV-PA coupling (low/uncoupled: SV/ESV < 0.80; mid: 0.80–1.14; high/coupled: ≥1.14), lung SUV did not differ significantly across groups (Kruskal–Wallis H = 3.74, *p* = 0.154). Median lung SUV was 0.47 [0.33–0.75] in the uncoupled group, 0.34 [0.29–0.40] in the mid group, and 0.40 [0.36–0.57] in the coupled group.

These findings indicate that SUV PA Proximal reflects the severity of pulmonary vascular disease (pressure load, resistance, vascular fibrosis), while SUV Lung reflects an independent metabolic process not coupled to hemodynamic severity.

### 3.3. Changes After PAH-Targeted Therapy

**Figure 2:**
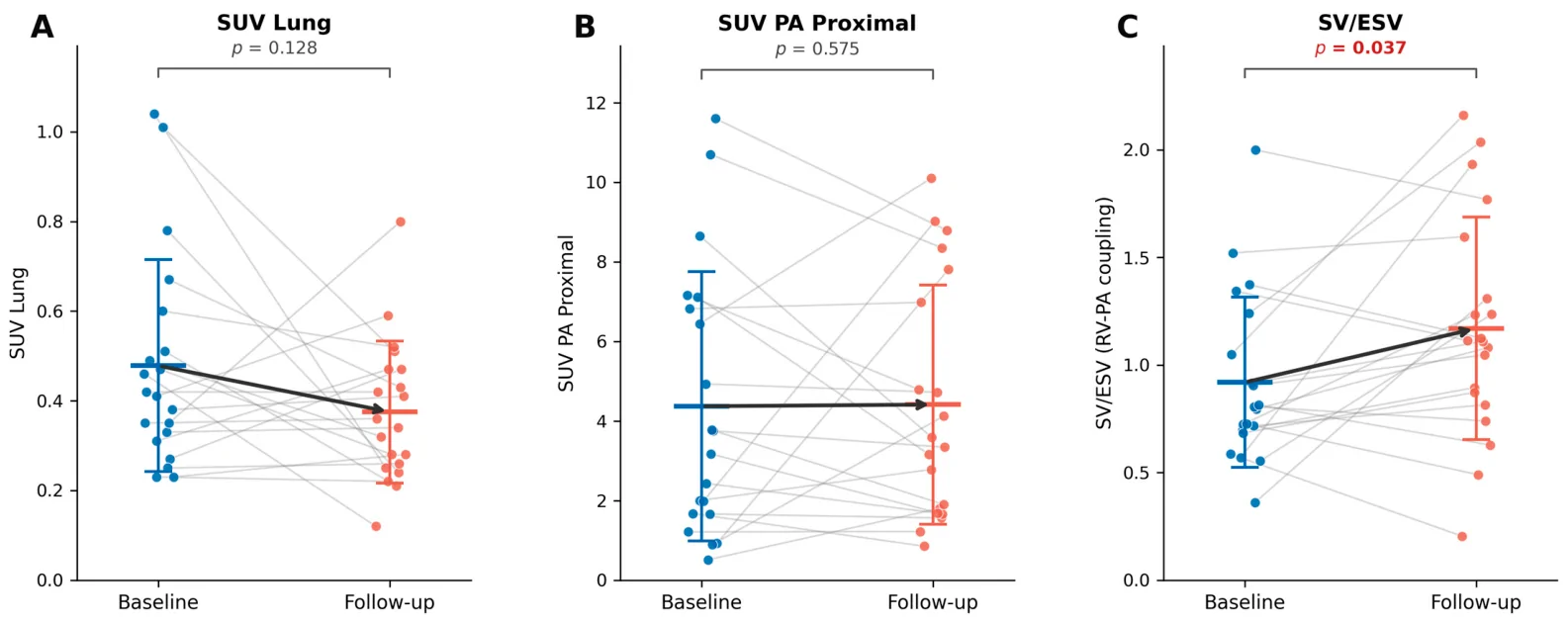
Paired changes after PAH-targeted therapy (N = 20). Slope graphs show individual patient trajectories from baseline to follow-up for (**A**) SUV Lung, (**B**) SUV PA Proximal and (**C**) SV/ESV (RV-PA coupling). ESV, end-systolic volume; PA, pulmonary artery; PAH, pulmonary arterial hypertension; SUV, standardized uptake value; SV, stroke volume.

Twenty PAH patients underwent repeat assessment after initiation or escalation of PAH-targeted therapy (Table 3). All CEP + patients had PAH therapy escalation between baseline and FU-1 visits—twelve patients started parenteral prostacycline (treprostinil or epoprostenol), one patient oral analogue (treprostinil), and three patients had added a second-line drug—macitentan. Both SUV parameters showed a non-significant trend toward reduction. The absence of treatment response suggests that proximal vascular wall metabolic activity is not meaningfully altered by currently available PAH therapies within the observed follow-up period. However, RHC-derived parameters, mPAP (50.6 ± 18.3 vs. 44.4 ± 19.6 mmHg, *p* = 0.04) and PVR (8.78 ± 5.82 vs. 6.79 ± 4.24 WU, *p* = 0.04), and especially RV-PA coupling surrogate—SV/ESV improved significantly (Figure 2). In contrast, functional capacity (6MWT: 404 ± 88 vs. 413 ± 78 m, *p* = 0.575) did not change significantly.

### 3.4. Survival Analysis and Prognostic Factors

**Figure 3:**
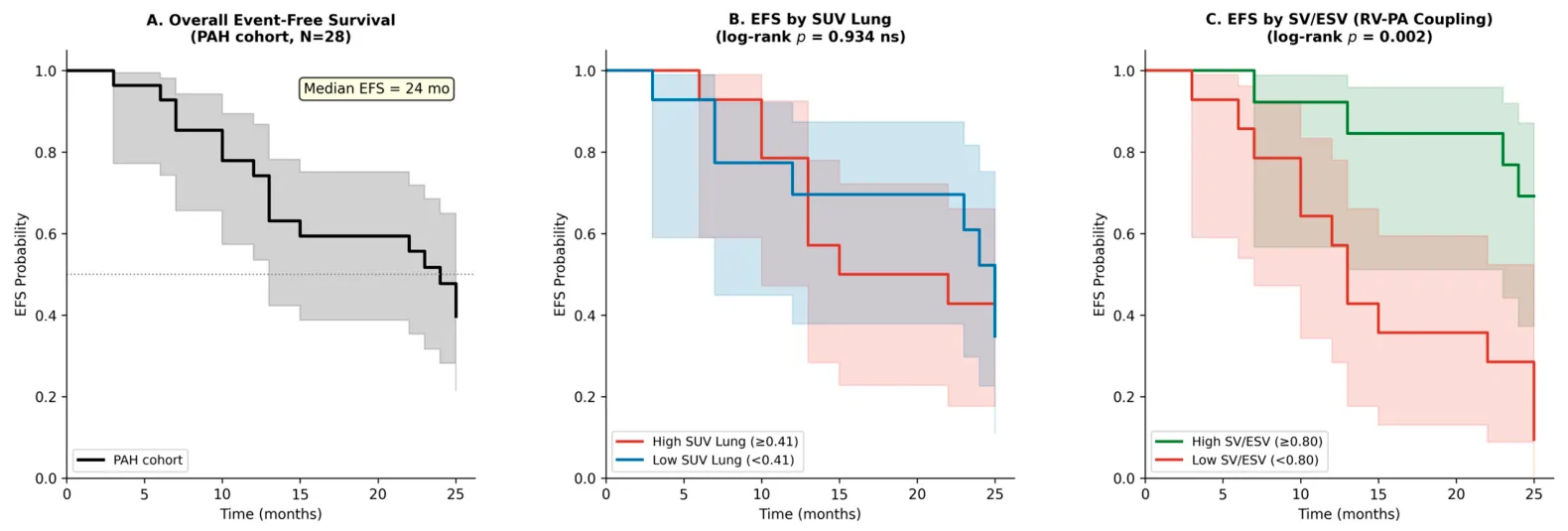
Kaplan–Meier event-free survival analysis. (**A**) Overall EFS in the PAH cohort (N = 28) with median EFS of 24 months. (**B**) EFS stratified by median lung SUV (0.41); no significant difference (log-rank *p* = 0.934). (**C**) EFS stratified by median SV/ESV (0.80); patients with low SV/ESV had significantly worse outcomes (log-rank *p* = 0.002). ESV, end-systolic volume; PA, pulmonary artery; PAH, pulmonary arterial hypertension; RV, right ventricle; SUV, standardized uptake value; SV, stroke volume.

Over a median follow-up of 22.5 months, 16 clinical endpoints (CEPs) occurred among the 28 PAH patients. The 12-month CEP was 74.3%, and the 24-month event-free survival (EFS) was 47.8%. SUV Lung and SUV PA Proximal did not predict CEP. When patients were stratified by median lung SUV (0.41) or PA proximal (3.46), Kaplan–Meier curves showed no difference in survival (log-rank χ^2^ = 0.007, *p* = 0.934; log-rank χ^2^ = 2.937, *p* = 0.086, respectively, Figure 3). In contrast, impaired RV-PA coupling was a strong predictor of adverse outcomes. Patients with SV/ESV < 0.80 (median split) had significantly worse EFS than those with SV/ESV ≥ 0.80 (log-rank χ^2^ = 9.90, *p* = 0.002; Figure 3).

In univariable Cox regression, only SUV PA Proximal was associated with events (HR = 1.15 [95% CI: 1.002–1.339], *p* = 0.047) alongside established predictors (mPAP, PVR, SV/ESV, RVEF), Table 4, but loses independent significance in the multivariable model (*p* = 0.107), consistent with collinearity with mPAP (r = +0.55).

Next, in multivariable Cox regression, including lung SUV, SV/ESV, and mPAP, lung SUV remained non-significant (HR = 0.51 [0.06–4.13], *p* = 0.532). SV/ESV showed a trend toward significance (HR = 0.20 [0.03–1.17], *p* = 0.073), while mPAP remained an independent predictor (HR = 1.05 [1.01–1.08], *p* = 0.011). The model achieved a concordance index of 0.847, indicating excellent discriminative ability.

## 4. Discussion

This study demonstrates an attempt to present the role of two distinct and independent metabolic signals detectable by^18^F-FDG PET/MRI: SUV Lung and SUV PA Proximal in PAH patients’ management and prognosis. Both metrics were markedly increased in PAH patients compared to age-matched healthy controls. Importantly, these two metabolic compartments were uncorrelated with each other (r = +0.105, *p* = 0.595), indicating that they reflect distinct pathophysiological processes within the pulmonary circulation. The potential role of cardiac FDG uptake in PAH was previously described but focused mostly on myocardial metabolic alterations [11,12,13].

The independence of parenchymal and vascular wall FDG shows that lung parenchymal FDG uptake likely reflects a combination of metabolic activity in small distal pulmonary vessels embedded within the parenchyma, perivascular inflammatory infiltrates, and potentially altered alveolar or interstitial cell metabolism in response to chronic vascular remodeling [10,13]. In contrast, proximal pulmonary artery wall FDG uptake represents direct metabolic activity within the large vessel wall itself, including proliferating smooth muscle cells, activated adventitial fibroblasts, and infiltrating inflammatory cells such as macrophages [14]. The spatial and cellular heterogeneity of these two compartments explains their statistical independence and suggests that they may respond differently to disease progression and therapeutic intervention.

Our findings extend prior observations of increased lung FDG uptake in PAH cohorts. Zhao et al. [13] reported heterogeneous lung FDG uptake in 20 PAH patients using dynamic PET with kinetic modeling, demonstrating increased signal in vascular compartments. Ohira et al. [10] found a mean lung FDG SUV of 0.76 ± 0.26 in PAH versus 0.53 ± 0.16 in controls (*p* = 0.0025), values comparable to our median SUV Lung of 0.405. Hagan et al. [14] provided proof-of-principle evidence for increased FDG uptake in both lungs and large pulmonary arteries in PH patients, linking uptake to immune activation markers. Our study confirms and extends these observations by simultaneously quantifying both compartments in the same patients and demonstrating their statistical independence and comparison to established hemodynamic parameters.

The pathophysiology of PAH involves progressive remodeling of the pulmonary arterial wall, characterized by endothelial dysfunction, smooth muscle cell proliferation, adventitial thickening, and inflammatory cell infiltration. These cellular processes are metabolically active and require increased glucose uptake to fuel proliferation, extracellular matrix synthesis, and inflammatory signaling. The correlation between SUV PA Proximal and hemodynamic parameters suggests that the metabolic activity of the vessel wall scales with the severity of hemodynamic burden, consistent with a dose-response relationship between pressure overload and vascular remodeling.

The biological basis for elevated FDG uptake in the pulmonary artery wall is multifactorial. Vascular imaging studies in atherosclerosis have established that arterial wall FDG uptake correlates with macrophage density and inflammatory activity. Importantly, Folco et al. demonstrated that hypoxia, rather than inflammation alone, is a major driver of glucose uptake in macrophages, suggesting that the hypoxic microenvironment within remodeled pulmonary artery walls may augment FDG signal [15]. Additionally, Marsboom et al. showed that pulmonary vascular cells in PAH undergo a glycolytic shift characterized by upregulation of Glut1 and increased glucose uptake, a metabolic phenotype analogous to the Warburg effect in cancer cells [16]. Thus, SUV PA Proximal likely reflects a combination of vascular cell glycolysis, inflammatory cell infiltration, and hypoxia-driven glucose uptake, all of which scale with hemodynamic severity.

In contrast to SUV PA Proximal, SUV Lung showed no significant correlations with any hemodynamic parameter after correction for multiple comparisons. Specifically, correlations between SUV Lung and mPAP, PVR, cardiac output, right atrial pressure, and all RV functional indices were non-significant after false discovery rate (FDR) correction. This lack of association indicates that parenchymal FDG uptake represents an independent metabolic signal that does not directly track with hemodynamic severity or RV adaptation in established PAH.

This finding is consistent with prior literature reporting inconsistent or absent correlations between lung FDG and hemodynamics. Ohira et al. found no correlation between mean lung FDG SUV and mPAP in PH patients [10], and Ruiter et al. reported no relationship between total lung SUV and PAH severity metrics in a cohort of treated IPAH patients [17]. Zhao et al. [13] observed marked intra-patient and inter-patient heterogeneity in lung FDG uptake, suggesting that parenchymal metabolic activity is spatially and temporally variable and may not uniformly reflect hemodynamic load. Our results confirm these observations in a well-characterized cohort with simultaneous invasive hemodynamics and cardiac MRI.

Several mechanisms may explain the independence of SUV Lung from hemodynamics. First, parenchymal FDG uptake likely reflects metabolic activity in small distal pulmonary vessels, perivascular inflammatory cells, and potentially altered alveolar or interstitial cell metabolism. These processes may be influenced by local factors, such as regional hypoxia, cytokine gradients, and cellular composition, which are not uniformly related to global hemodynamic parameters. Second, the metabolic signature of the lung parenchyma may be heterogeneous, with some regions exhibiting high FDG uptake due to active vascular remodeling or inflammation, while other regions remain relatively quiescent. This spatial heterogeneity, captured by Zhao et al. using dynamic PET, may obscure correlations with global hemodynamic measures. Third, technical factors such as partial volume effects, respiratory motion, and variability in ROI placement may introduce noise into SUV Lung measurements, reducing their correlation with hemodynamic parameters.

After initiation of PAH-targeted therapy, SUV Lung and SUV PA Proximal exhibited strikingly divergent responses, further supporting their biological independence. SUV Lung showed a non-significant trend toward reduction (Δ = −0.103, −21.5%, *p* = 0.128), whereas SUV PA Proximal remained completely unchanged (Δ = +0.04, +1%, *p* = 0.575). In contrast, RV-PA coupling (SV/ESV) improved significantly (+27.4%, *p* = 0.037), indicating favorable RV reverse remodeling and improved mechanical efficiency of the RV-arterial system.

The modest and non-significant reduction in SUV Lung suggests that parenchymal metabolic activity may be less responsive to short-term hemodynamic improvement than RV functional parameters. This finding is consistent with Ruiter et al., who observed relatively low lung FDG uptake in treated IPAH patients and reported no relationship with severity metrics, suggesting that chronic therapy may partially normalize parenchymal metabolism but that residual metabolic abnormalities persist [18]. Marsboom et al. also demonstrated that lung and vascular FDG uptake was reversible with targeted therapies in animal models, supporting the potential for metabolic normalization with effective treatment [16]. However, the time course and magnitude of metabolic response in humans may differ from animal models, and a longer treatment duration or larger sample size may be needed to detect significant changes in SUV Lung.

The significant improvement in RV-PA coupling without corresponding changes in SUV PA Proximal further supports the notion that these measures capture different aspects of PAH pathophysiology. RV-PA coupling reflects the mechanical efficiency of the RV-arterial system, which can improve with afterload reduction and RV reverse remodeling even in the absence of vascular metabolic normalization [8,18]. This dissociation suggests that hemodynamic and functional improvements can occur independently of metabolic changes in the pulmonary artery wall, and that metabolic imaging may provide complementary information about the biological state of the pulmonary vasculature beyond what is captured by hemodynamics and RV function.

This leads us to the importance of RV-PA coupling, quantified by the ratio of stroke volume to end-systolic volume (SV/ESV). It has emerged as the dominant prognostic marker in our cohort, far outperforming both FDG metabolic metrics and individual hemodynamic parameters. PAH patients exhibited severely impaired RV-PA coupling compared to controls (0.800 vs. 1.680, *p* < 0.001). In survival analysis, low SV/ESV was strongly associated with adverse outcomes (Kaplan–Meier log-rank *p* = 0.002; Cox hazard ratio [HR] = 0.04 [95% CI: 0.006–0.30], *p* = 0.002). In multivariable Cox regression adjusting for mPAP, SV/ESV remained the dominant predictor with a C-index of 0.847, indicating excellent prognostic discrimination.

These findings are consistent with a robust body of literature establishing RV-PA coupling as a powerful integrative measure of RV-arterial interaction and a strong predictor of outcomes in PAH. Vanderpool et al. demonstrated that SV/ESV was an independent predictor of transplant-free survival in 50 PH patients after adjustment for right atrial pressure, mPAP, and stroke volume [8]. Swift et al. identified RV end-systolic volume index (RVESVI) as an independent MRI predictor of death in 576 PAH patients in the ASPIRE registry, with 221 deaths over a median follow-up of 42 months [19]. Hsu et al. showed that impaired RV-arterial coupling predicts clinical worsening in PAH using multi-beat invasive coupling measures [20], and Nakaya et al. similarly reported that RV-PA uncoupling predicts poor outcomes in PAH cohorts [21].

Proposed numeric thresholds for SV/ESV vary across studies and modalities. Zhu et al. found that SV/ESV ≤ 0.55 predicted adverse outcomes in connective tissue disease-associated PAH patients and that adding SV/ESV improved prognostic models [22]. In our study, we used the median SV/ESV value; in contrast to the cited study [22], patients with CTD-PAH (a subtype often with a poorer prognosis) constituted only 18%.

In this manuscript, we showed that neither SUV Lung nor SUV PA Proximal demonstrated independent prognostic value in our cohort. In contrast, established hemodynamic and RV functional parameters demonstrated strong prognostic associations. It is important to note that our findings pertain specifically to lung parenchymal and proximal pulmonary artery wall FDG uptake and do not contradict prior literature on RV myocardial FDG uptake. Our current study did not focus on RV myocardial uptake, and thus our findings do not address the potential prognostic value of RV metabolic imaging. In previous papers, we proved the importance of single SUV RV/LV assessment of PAH patients [3,4].

However, our findings may have some clinical implications. First,^18^F-FDG PET/MRI can reliably distinguish PAH patients from healthy controls based on both parenchymal and vascular metabolic activity, supporting its potential utility as a diagnostic tool. The large effect sizes for both SUV Lung (r = 0.866) and SUV PA Proximal (r = 0.670) indicate robust discrimination between PAH and controls.

Secondly, results support the potential role of SV/ESV as an additional parameter to assess the hemodynamic status of PAH patients. Results showed its prognostic discrimination (C-index = 0.847) and its responsiveness to therapy; thus, it may be a novel helpful tool for monitoring treatment response and guiding escalation of therapy.

Finally, the importance of SUV Lung and SUV PA Proximal, and their divergent relationships with hemodynamics and treatment response, suggest that PAH involves multiple distinct pathophysiological processes that may require different therapeutic approaches. A precision medicine approach that phenotypes patients based on metabolic, hemodynamic, and RV functional profiles may enable more targeted and effective therapy. For example, patients with high SUV PA Proximal and severe hemodynamic impairment may benefit from aggressive vasodilator therapy combined with anti-inflammatory agents, whereas patients with high SUV Lung but preserved hemodynamics may benefit from therapies targeting parenchymal inflammation or distal vascular remodeling.

### Study Limitations

This study has several limitations that should be acknowledged. First, the sample sizes of both control and study groups were modest, particularly for the treatment response and survival analyses in the PAH group, which may have limited statistical power to detect associations between FDG metrics and outcomes. Larger, multicenter cohorts are needed to definitively establish the prognostic value of metabolic PET imaging in PAH. Second, we used static SUV measurements rather than dynamic PET with kinetic modeling [22]. Third, FDG is a non-specific tracer that reflects glucose uptake in all metabolically active cells, including vascular cells, inflammatory cells, and potentially parenchymal cells. The biological specificity of FDG uptake is limited, and mechanistic attribution requires correlative tissue or cell-specific studies. Fourth, technical factors such as partial volume effects, blood pool activity, and respiratory motion may introduce variability into SUV measurements, particularly for small structures such as the proximal pulmonary artery wall. Future studies should employ dynamic PET to more precisely characterize metabolic activity in different pulmonary compartments and to assess whether kinetic parameters provide incremental prognostic information beyond static SUV (Zhao et al. demonstrated that dynamic FDG-PET with kinetic analysis reveals significant intra-lung metabolic heterogeneity in PAH that is not captured by static SUV alone [13]. Finally, the multivariable Cox model included three covariates against 16 events; thus, readers should interpret the hazard ratio estimates with caution, and independent validation in larger prospective cohorts is essential before clinical translation.

## 5. Conclusions

Integrated FDG-PET/MRI demonstrates significantly elevated glucose metabolism in both the lung parenchyma (SUV Lung) and the proximal pulmonary artery wall (SUV PA Proximal) in PAH patients compared with healthy controls, confirming active metabolic inflammation across distinct vascular compartments Table 5. Despite haemodynamic improvement following PAH-targeted therapy, neither SUV Lung nor SUV PA Proximal changed significantly over the follow-up period, suggesting that metabolic inflammation within the pulmonary vasculature and parenchyma persists beyond pharmacological haemodynamic control. SUV PA Proximal was a statistically significant univariable predictor of clinical events (HR 1.16, 95% CI 1.00–1.34; *p* = 0.047) yet did not retain independent prognostic value in multivariable models, where mPAP emerged as the dominant haemodynamic predictor. Future research should explore the biological basis of elevated pulmonary FDG uptake, its evolution over time, and whether targeted therapies can modulate pulmonary metabolic activity and improve outcomes.

## Figures and Tables

**Table 1:** Baseline characteristics, SUV metrics and hemodynamic function of PAH patients and healthy controls.

	PAH (*n* = 28)	Control (*n* = 12)	*p*-Value
Age (years)	51.4 ± 15.9	44.8 ± 13.5	0.190
Sex (females)	17 (60)	8 (67)	0.567
PAH Etiology			
Idiopathic PAH	19 (68)	—	—
CTD-PAH	5 (18)	—	—
CHD-PAH	3 (11)	—	—
Heritable PAH	1 (4)	—	—
WHO Functional Class			
Class I	4 (14)	—	—
Class II	12 (43)	—	—
Class III	12 (43)	—	—
SUV (mean) Metrics			
SUV Lung	0.405 [0.338–0.533]	0.225 [0.207–0.273]	<0.001
SUV PA Proximal	3.46 [1.95–6.89]	1.48 [1.15–1.65]	<0.001
RV Volumes and Function (MRI)			
RV EDV (mL)	219 [181–251]	136 [116–157]	<0.001
RV ESV (mL)	122 [94–141]	50 [42–63]	<0.001
RV EF (%)	44.3 ± 10.1	63.8 ± 5.9	<0.001
SV/ESV (coupling)	0.80 [0.69–1.14]	1.68 [1.43–1.92]	<0.001
TAPSE (mm)	18.5 [15.8–22.3]	25.0 [23.8–27.0]	<0.001
RV GLS (%)	−18.0 ± 8.0	−31.5 ± 9.7	<0.001
RV Remodeling			
RV wall thickness (mm)	6.0 [4.0–7.0]	2.5 [2.4–3.0]	<0.001
RA area (cm^2^)	25.3 [22.6–31.2]	21.0 [16.5–21.8]	<0.001
Pulmonary trunk (mm)	36.5 [32.0–40.0]	23.0 [19.5–27.3]	<0.001

Data presented as mean ± SD or median [IQR]. Categorical variables are presented as numbers (percentages). CHD, congenital heart disease; CTD, connective tissue disease; EDV, end-diastolic volume; EF, ejection fraction; ESV, end-systolic volume; GLS, global longitudinal strain; MRI, magnetic resonance imaging; *n*, number; PA, pulmonary artery; PAH, pulmonary arterial hypertension; RA, right atrium; RV, right ventricle; SUV, standardized uptake value; SV, stroke volume; TAPSE, tricuspid annular plane systolic excursion; WHO, World Health Organization.

**Table 2:** Spearman correlations between SUV and hemodynamic/functional parameters in PAH patients.

	SUV Lung		SUV PA Proximal	
Variable	Spearman r	*p*-Value	Spearman r	*p*-Value
mPAP (mmHg)	−0.021	0.917	+0.551	<0.001
PAPs (mmHg)	−0.094	0.634	+0.518	0.002
PAPd (mmHg)	0.033	0.868	+0.529	0.002
PVR (WU)	−0.061	0.760	+0.517	0.002
CO (L/min)	−0.155	0.430	−0.304	0.104
CI (L/min/m^2^)	−0.069	0.726	−0.119	0.541
PCWP (mmHg)	0.215	0.271	+0.051	0.796
CVP (mmHg)	0.074	0.707	+0.377	0.038
RV EF (%)	−0.005	0.982	−0.288	0.125
RV EDV (mL)	0.030	0.881	+0.174	0.367
RV ESV (mL)	−0.001	0.997	+0.191	0.321
SV/ESV (coupling)	−0.005	0.980	−0.221	0.248
TAPSE (mm)	0.194	0.321	−0.065	0.739
RV GLS (%)	−0.023	0.913	+0.203	0.321
BNP (pg/mL)	0.040	0.842	+0.455	0.009
6MWT (m)	−0.056	0.778	−0.235	0.217
PT diameter (mm)	−0.354	0.064	+0.270	0.153
RA area (cm^2^)	−0.047	0.814	+0.108	0.579

6MWT, six-minute walk test; BNP, brain natriuretic peptide; CI, cardiac index; CO, cardiac output; CVP, central venous pressure; EDV, end-diastolic volume; EF, ejection fraction; ESV, end-systolic volume; GLS, global longitudinal strain; mPAP, mean pulmonary arterial pressure; PA, pulmonary artery; PAH, pulmonary arterial hypertension; PAPd, pulmonary arterial pressure diastolic; PAPs, pulmonary arterial pressure systolic; PCWP, pulmonary capillary wedge pressure; PT, pulmonary trunk; PVR, pulmonary vascular resistance; RA, right atrium; RV, right aentricle; SUV, standardized uptake value; SV, stroke volume; TAPSE, tricuspid annular plane systolic excursion; WU, wood units.

**Table 3:** Paired changes in SUV, hemodynamics, and RV function after PAH-targeted therapy.

Variable	Baseline	Follow-Up	Δ (Change)	*p*-Value
SUV Lung	0.478 ± 0.236	0.375 ± 0.158	−0.103 (−5.9%)	0.128
SUV PA Proximal	4.37 ± 3.38	4.41 ± 3.01	+0.04 (+1%)	0.575
mPAP (mmHg)	50.6 ± 18.3	44.4 ± 19.6	−6.2 (−10.1%)	0.042
PVR (WU)	8.78 ± 5.82	6.79 ± 4.24	−1.99 (−14.1%)	0.043
SV/ESV (coupling)	0.92 ± 0.40	1.17 ± 0.52	+0.25 (+27.4%)	0.037
RV EF (%)	45.8 ± 9.2	51.2 ± 12.7	+5.4 (+14.9%)	0.049
CO (L/min)	4.83 ± 1.14	5.23 ± 1.17	+0.40 (+10.8%)	0.057
6MWT (m)	404 ± 88	413 ± 78	+8.6 (+3.6%)	0.575
TAPSE (mm)	19.1 ± 4.6	17.8 ± 6.4	−1.3 (−6.9%)	0.234

Data presented as mean ± SD. 6MWT, six-minute walk test; CO, cardiac output; EF, ejection fraction; mPAP, mean pulmonary arterial pressure; PA, pulmonary artery; PVR, pulmonary vascular resistance; RV, right ventricle; SD, standard deviation; SUV, standardized uptake value; SV, stroke volume; TAPSE, tricuspid annular plane systolic excursion; WU, wood units.

**Table 4:** Cox proportional hazards regression for event-free survival.

Variable	Univariable Hazard Ratio	95% CI	*p*-Value	Multivariable Hazard Ratio	*p*-Value
SUV Lung	0.42 (0.04–4.90)	0.04–4.90	0.486	0.51 (0.06–4.13)	0.532
SUV PA Proximal	1.15 (1.00–1.33)	1.00–1.33	0.047		
SV/ESV (coupling)	0.04 (0.01–0.30)	0.01–0.30	0.002	0.20 (0.03–1.17)	0.073
mPAP (mmHg)	1.08 (1.04–1.12)	1.04–1.12	<0.001	1.05 (1.01–1.08)	0.011
PVR (WU)	1.15 (1.05–1.25)	1.05–1.25	0.002		
RV EF (%)	0.91 (0.87–0.96)	0.87–0.96	<0.001		
TAPSE (mm)	0.91 (0.80–1.03)	0.80–1.03	0.132		
6MWT (m)	0.995 (0.991–1.000)	0.991–1.000	0.032		

6MWT, six-minute walk test; CI, confidence interval; EF, ejection fraction; HR, hazard ratio; mPAP, mean pulmonary arterial pressure; PA, pulmonary artery; PVR, pulmonary vascular resistance; RV, right ventricle; SUV, standardized uptake value; SV, stroke volume; TAPSE, tricuspid annular plane systolic excursion; WU, wood units.

**Table 5:** Comparative summary: SUV PA Proximal vs. SUV Lung.

Feature	SUV PA Proximal	SUV Lung
Biological compartment	Vascular wall	Lung parenchyma
Elevated in PAH vs. control	✓ Yes (*p* < 0.001)	✓ Yes (*p* < 0.001)
Correlates with mPAP/PVR	✓ Yes (r ≈ 0.52–0.55)	✗ No
Changes with treatment	✗ No (*p* = 0.953)	Trend (*p* = 0.128)
Predicts outcomes (univariable)	Trend (*p* = 0.057)	✗ No (*p* = 0.486)
Independent predictor	✗ No	✗ No
C-index	0.622	0.528

mPAP, mean pulmonary arterial pressure; PA, pulmonary artery; PAH, pulmonary arterial hypertension; PVR, pulmonary vascular resistance; SUV, standardized uptake value; SV, stroke volume.

## Data Availability

The datasets generated and/or analyzed during the study are available from the corresponding author upon reasonable request.

## Abbreviations

The following abbreviations are used in this manuscript:

^18^F-FDG	^18^F-Fluorodeoxyglucose
6MWT	Six-Minute Walk Test
BNP	Brain Natriuretic Peptide
C-index	Concordance Index
CEP	Clinical Endpoint
CHD	Congenital Heart Disease
CI	Cardiac Index / Confidence Interval
CO	Cardiac Output
CTD	Connective Tissue Disease
CVP	Central Venous Pressure
EDV	End-Diastolic Volume
EF	Ejection Fraction
EFS	Event-Free Survival
ESV	End-Systolic Volume
FDG	^18^F-Fluorodeoxyglucose
FDR	False Discovery Rate
GLS	Global Longitudinal Strain
Glut1	Glucose Transporter 1
hPAH	Heritable Pulmonary Arterial Hypertension
HR	Hazard Ratio
IPAH	Idiopathic Pulmonary Arterial Hypertension
IQR	Interquartile Range
LGE	Late Gadolinium Enhancement
LV	Left Ventricle
mPAP	Mean Pulmonary Arterial Pressure
MRI	Magnetic Resonance Imaging
MV	Multivariable
PA	Pulmonary Artery
PAH	Pulmonary Arterial Hypertension
PAPd	Pulmonary Arterial Pressure, Diastolic
PAPs	Pulmonary Arterial Pressure, Systolic
PCWP	Pulmonary Capillary Wedge Pressure
PET	Positron Emission Tomography
PET/MRI	Positron Emission Tomography/Magnetic Resonance Imaging
PH	Pulmonary Hypertension
PT	Pulmonary Transit Time
PVR	Pulmonary Vascular Resistance
RA	Right Atrium
ROI	Region of Interest
RV	Right Ventricle
RVEF	Right Ventricular Ejection Fraction
RVESVI	Right Ventricular End-Systolic Volume Index
SD	Standard Deviation
SUV	Standardized Uptake Value
SV	Stroke Volume
SV/ESV	Stroke Volume to End-Systolic Volume Ratio (RV-PA Coupling Index)
TAPSE	Tricuspid Annular Plane Systolic Excursion
WHO	World Health Organization
WU	Wood Units
